# Aberrant Neural Response to Social Exclusion Without Significantly Greater Distress in Youth With Bipolar Disorder: Preliminary Findings

**DOI:** 10.3389/fpsyt.2022.687052

**Published:** 2022-04-01

**Authors:** Donna J. Roybal, Victoria E. Cosgrove, Ryan Kelley, Rachel Smallwood Shoukry, Rose Marie Larios, Blake Novy, Kiki D. Chang, Amy S. Garrett

**Affiliations:** ^1^Division of Child and Adolescent Psychiatry, Department of Psychiatry and Behavioral Sciences, Stanford University School of Medicine, Stanford, CA, United States; ^2^Center for Interdisciplinary Brain Sciences Research, Department of Psychiatry and Behavioral Sciences, Stanford University School of Medicine, Stanford, CA, United States; ^3^Research Imaging Institute, University of Texas Health Science Center, San Antonio, TX, United States; ^4^Department of Psychiatry and Behavioral Sciences, University of Texas Health Science Center, San Antonio, TX, United States

**Keywords:** bipolar, social exclusion, neuroimaging, anxiety, cyberball

## Abstract

**Background:**

Little is known about the effects of social exclusion on youth with bipolar disorder (BD). Understanding these effects and the functional neural correlates of social exclusion in youth with BD may establish differences from healthy youth and help identify areas of intervention.

**Methods:**

We investigated brain function in 19 youth with BD and 14 age and gender matched healthy control (HC) participants while performing Cyberball, an fMRI social exclusion task. Whole brain activation, region-of-interest, and functional connectivity were compared between groups and examined with behavioral measures.

**Results:**

Compared with the HC group, youth with BD exhibited greater activation in the left fusiform gyrus (FFG) during social exclusion. Functional connectivity between the left FFG and the posterior cingulate/precuneus was significantly greater in the HC compared with the BD group. For the HC group only, age and subjective distress during Cyberball significantly predicted mean FFG activation. No significant differences in distress during social exclusion were found between groups.

**Conclusion:**

Although preliminary due to small sample size, these data suggest that youth with BD process social exclusion in a manner that focuses on basic visual information while healthy youth make use of past experiences to interpret current social encounters. This difference may account for the social cognitive issues experienced by youth with BD, which can lead to more severe anxiety and mood symptoms.

## Introduction

Bipolar disorder (BD) with comorbid anxiety is associated with poorer response to treatment, more severe depression, rapid cycling, substance abuse, and suicide attempts (1–5). Emerging longitudinal evidence suggests that youth at high-risk for BD that develop any mood disorder experience an anxiety disorder as an early antecedent (2, 5, 6). In fact, the risk of a mood disorder diagnosis was over two times higher in those with an anxiety disorder than those without, with social anxiety disorder and generalized anxiety disorder the most predictive (5). Anxiety is therefore an important symptom in the developmental trajectory of BD, both as a comorbidity and as a potential risk factor for the development of a mood disorder.

One of the largest sources of anxiety in youth is the quality of social relationships, which greatly influence youth’s perceived quality of life (7–9). Youth with BD demonstrate deficits in interpersonal functioning that contribute to anxiety (9, 10) and undermine emotion regulation, potentially leading to mood episodes (10–13). A major source of anxiety for youth is social exclusion (14). Therefore, better understanding of responses to social exclusion in youth with BD could lead to interventions that prevent mood symptom development.

No previous studies have examined the neural underpinnings of social exclusion in youth with BD. For youth with unipolar depression, previous studies of social exclusion have reported abnormal hyperactivation of the anterior insula and subgenual cingulate cortex (sgACC) (11, 12, 15–18), which was correlated with greater feelings of distress compared with healthy controls (HC) (12). Additionally, hyperactivation in the sgACC and medial prefrontal cortex (PFC) during social exclusion was predictive of depressive symptoms one year later (13). FMRI studies also suggest the ventral PFC and ventral striatum regulate areas hyperactivated during social exclusion (12, 15). In fact, a recent coordinate-based meta-analysis found that activation in the right ventral striatum and left ventrolateral prefrontal cortex (VLPFC) is consistently reported in studies of developmental samples during a social exclusion fMRI task called “Cyberball” (19). Taken together, these studies suggest that the neural response to social exclusion involves structures associated with internal perception (anterior insula) and emotional experience (sgACC) regulated by the VLPFC. We therefore hypothesized that youth with BD would exhibit greater activation of this neural circuitry and report significantly greater distress during social exclusion when compared with HC.

## Materials and Methods

### Participants and Assessments

The Stanford University Administrative Panel of Medical Research in Human Subjects approved the protocol. We recruited 19 youth fulfilling DSM-IV-TR criteria for BD I, II, or not otherwise specified (NOS) from a pediatric bipolar disorders clinic and 16 gender and age matched healthy controls (HC) from the surrounding community. We examined the bipolar spectrum of disease owing to the fact that longitudinal studies have shown that within 2.5 years, youth with BD, NOS convert to BD II or I, and youth with BD II convert to BD I (1). All participants were between the ages of 10–18. We obtained written informed consent and assent from the parents and children, respectively. Children were administered the Young Mania Rating Scale (YMRS) (20) and the Children’s Depression Rating Scale-Revised Version (CDRS-R) (21) by raters with established inter-rater reliability (ICC > 0.9). Participants were also administered the children’s Rejection Sensitivity Questionnaire (RSQ), a validated scale measuring the severity of anxiety and anger that might be experienced with regards to the likelihood of being accepted in various social exclusion scenarios (22). Participants were also administered the Need Threat Scale (NTS), a validated scale used to assess the severity of subjective distress felt during the fMRI social exclusion task (23, 24). Subjective distress, as defined by the NTS, assesses the degree of threat someone feels during social exclusion to their needs for belonging, control, self-esteem, and meaningful existence (23, 24). The NTS is scored such that higher scores indicate lower levels of subjective distress, or threat to need, and lower scores indicate higher subjective distress.

The affective module of the Washington University in St. Louis Kiddie-Schedule for Affective Disorders and Schizophrenia (WASH-U KSADS) (kappa > 0.9 for diagnostic reliability) (25, 26) and the Kiddie–Schedule for Affective Disorders and Schizophrenia, Present and Lifetime (kappa 0.77–1.00 for diagnostic reliability) (27) were administered to parents and children in separate interviews by a trained masters-level clinician and/or board-certified psychiatrist. DSM-IV-TR criteria were used to determine current and lifetime psychiatric diagnoses. BD-NOS criteria was defined as a minimum of either (1) two lifetime episodes of at least four hours duration each of criterion A: either elevated mood plus two associated symptoms or irritable mood plus three associated symptoms, but not meeting threshold BD I or II criteria or (2) 2–3 days of criterion A. Participants taking medications were stable on medications, defined as three weeks at the same dosage if taking a selective serotonin reuptake inhibitor (SSRI), and 2 weeks if taking a mood stabilizer, antipsychotic, and/or stimulant.

Youth were excluded from the BD group if they had diagnoses of pervasive development disorder, intellectual disability, obsessive-compulsive disorder, panic disorder, post-traumatic stress disorder, a history of head trauma with loss of consciousness, or Tourette’s syndrome. Participants in the healthy control group were excluded if they were taking psychotropic medications or if they or any of their first-degree relatives had a current or lifetime DSM-IV-TR diagnosis. Further excluded from either group were any children with a neurologic condition (e.g., seizure disorder), substance use disorder, or the presence of metallic implants or braces.

### “Cyberball” Task During fMRI

Participants were scanned while playing Cyberball, a computer game used to study the effects of social exclusion that has been adapted for use in the fMRI scanner (23, 24, 28). In this game, the participant played a virtual ball-tossing game with two other players. To enhance the interpersonal nature of the game, the participant was told s/he was playing with two other players and that each player was in a separate scanner. These two other players were shown as cartoon figures on the projection screen viewed by the participant via a mirror attached to the headcoil. The participant was represented by a cartoon hand at the bottom of the screen.

The cyberball task was designed to replicate that used in previous studies (12). The task is a block design containing “inclusion” and “exclusion” blocks. During inclusion blocks, a cartoon ball was thrown to the participant, who could then throw the ball to one of the two other (cartoon) players by pressing the left or right button on the button box. During exclusion blocks, the ball was thrown to one of the other (cartoon) players, and the participant was excluded from all throws. For all blocks, each throw had a duration of 5–6 s (depending on how quickly the participant threw the ball) with an inter-throw interval of 0.5 s. The order of blocks was inclusion–inclusion–exclusion. The first inclusion block contained 55 throws, the second inclusion block contained 30 throws, and the exclusion block contained 27 throws. Overall, the task duration was 4:29”.

After the scan, participants were administered the Need Threat Scale to assess the severity of subjective distress experienced during the game (23, 24).

### fMRI Acquisition and Preprocessing

Magnetic resonance imaging scans were conducted at the Stanford University Richard M. Lucas Center for Imaging. Images were acquired using a 3.0T General Electric MR750 scanner (General Electric, Milwaukee, WI, United States) using an 8-channel head coil. The following pulse sequence parameters were used for the fMRI scans: spiral in-out, echo time (TE)/repetition time (TR) = 30/2,000 ms, flip angle = 89° and 1 interleave, matrix size 64 × 64, field of view (FOV) = 240 mm, 31 slices, slice thickness 4 mm, skip 0.5 mm; entire brain and cerebellum. An individually calculated high-order shim for spiral acquisitions was used to reduce field inhomogeneity. A high resolution fast spoiled grass (FSPGR) anatomical scan also was collected to optimize registration of fMRI data to standard space.

### fMRI Data Preprocessing

Functional MRI data were analyzed using SPM8 software.^1^ Images were realigned to the third volume and motion was corrected using the ArtRepair toolbox (cibsr.stanford.edu/tools/ArtRepair). Volumes with motion artifact (slope > 1.5 mm/volume) were replaced with a volume that was interpolated from the nearest surrounding unaffected volumes. Scans were rejected from further analysis for motion spikes greater than 4 mm translation or if more than 20% of volumes required motion correction. Images were normalized to the MNI152 template using each subject’s anatomical scan and resampled to a 2-cubic mm matrix using sinc interpolation, smoothed with a 5 mm FWHM Gaussian filter, and high pass filtered at 120 s.

### Group Differences in Ratings of Distress and Rejection Sensitivity

An independent-sample *t*-test performed in IBM SPSS v26.0^2^ was used to examine differences between BD and HC group mean NTS scores. Spearman’s correlations within each group were used to examine the association between NTS and RSQ scores. RSQ scores comprised two scores, an anger and an anxiety domain. NTS scores for each group were therefore correlated with each domain separately. Thresholds for significance were set at q = 0.05, after FDR correction for multiple comparisons.

### Whole Brain Analyses

For each subject, a fixed-effects analysis in SPM8 using the general linear model was performed to calculate voxel-wise statistical maps for each subject, for the contrast of exclusion minus inclusion blocks. Between group voxel-wise comparisons were conducted using an independent groups t-test, while covarying for age.

Inference was conducted using a cluster-forming threshold of *p* < 0.005, combined with family-wise error correction of *p* < 0.05 at the cluster level. While our cluster-forming threshold of *p* = 0.005 is somewhat more liberal than the traditional setting of *p* = 0.001 (29), it is recommended for reducing Type II error in fMRI studies of social and affective processes, which have small effect sizes and weak statistical power due to the complexity of these psychological processes (30,31). In addition, this threshold is similar to previous studies examining the effects of Cyberball in youth (19, 30). Age was covaried given previous findings that brain regions activated by Cyberball were age dependent (19).

### Region-of-Interest Analyses

Regions of interest (ROI) were defined using the Automated Anatomic Labeling (AAL) atlas (32) for the anterior insula and the anterior cingulate cortex. For the ventral striatum and ventral PFC, coordinates were taken from a meta-analysis of developmental Cyberball studies and a 5 mm sphere was created for each *a priori* region using MarsBar^3^ from which mean activation was extracted (19).

### Functional Connectivity

The generalized Psychophysiological Interaction (gPPI) toolbox (33) was used to examine whole brain functional connectivity with seeds placed at each of the significant activation clusters. The resulting voxel-wise connectivity maps were contrasted between the BD and HC groups using independent groups *t*-tests in SPM8. Inference was conducted using a cluster-forming threshold of *p* < 0.005, combined with Family-wise error correction of *p* < 0.05 at the cluster level, as justified in the section “Whole Brain Analyses.”

### Associations Between Significant Clusters and Self-Reported Distress and Mood Symptomatology

Within each group separately, linear regression in SPSS was used to predict NTS score from mean activation in each significant cluster, adjusted for age. A second model was used to predict RSQ from mean activation in significant clusters.

CDRS-R scores measuring depression symptoms were correlated with each individual’s mean activation for each significant cluster using Spearman’s rho. Thresholds for significance were set at q = 0.05, after FDR correction for multiple comparisons. The same was performed for mania symptoms using YMRS scores.

Whole-brain linear regression was also performed for each group twice using activation and functional connectivity each as dependent variables in separate models in SPM8. Total NTS scores and age were the independent variables in these models. We used a cluster forming threshold of p = 0.005 and thresholds of inference set at p ≤ 0.05, FWE corrected.

## Results

### Demographics and Clinical Characteristics

Two HC scans were not usable, one due to artifact during the exclusion run and the second due to incomplete capture of superior portions of the brain. A total of 19 scans in the BD group and 14 scans in the HC group were included in fMRI analysis. There were no group differences in age [t(30) = 0.540, *p* = 0.74] or proportion of females to males (χ^2^ = 0.07, *p* = 0.80). Nine youth were diagnosed with BD I and ten with BD, NOS. Four had Generalized Anxiety Disorder and one had Oppositional Defiant Disorder. Table 1 provides additional demographic and clinical characteristics.

**TABLE 1:** Description of participants.

	Bipolar Group (*n* = 19)	Healthy Control Group (*n* = 14)	Group comparison *p*-value
Males	10 (53%)	8 (57%)	0.066
Females	9 (47%)	6 (43%)	0.797
Age (mean ± SD)	14.97 ± 2.00	14.73 ± 2.07	0.740
Body Mass Index (BMI)	24.0 ± 5.0	20.4 ± 1.5	0.086
Motion during MRI scan (absolute displacement in mm)	0.12 ± 0.10	0.14 ± 0.15	0.753
**Primary Diagnosis**			
Bipolar I	9 (47%)	None	
Bipolar, NOS	10 (53%)		
Comorbid Diagnoses		None	
Generalized Anxiety Disorder	4 (21%)		
ODD^4^	1 (5%)		
% Taking or Exposed to Meds	53%	None	
**Medication at Time of Scan**		None	
Antidepressants (SSRI)	5%		
Stimulant	16%		
Lithium	11%		
Other mood stabilizers	21%		
Antipsychotics	32%		
Anxiolytics	0%		

*NOS, not otherwise specified; ODD, Oppositional Defiant Disorder; SSRI, Selective Serotonin Reuptake Inhibitors.*

### Group Differences in Mood Symptoms and Distress During Exclusion

**FIGURE 1:**
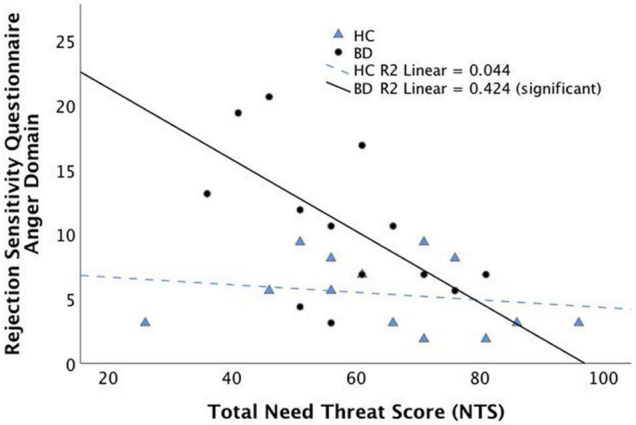
Spearman’s correlation between subjective distress during social exclusion (as measured by the Need Threat Scale) and the anger domain of the Rejection Sensitivity Questionnaire (RSQ). Correlation is significant within the Bipolar Disorder group but not the Healthy Control group.

Table 2 depicts CDRS-R, YMRS, NTS, and RSQ scores for each group. No significant difference was found between the BD and HC groups for mean NTS scores (*p* = 0.33). The BD group had significantly higher scores when compared with HC for the anger domain [t(24) = 2.73, *p* = 0.012] and the anxiety domain [t(28) = 2.15, *p* = 0.041] of the RSQ. As expected, YMRS and CDRS-R scores were significantly higher in the BD group [YMRS: t(21) = 3.04, *p* = 0.006; CDRS-R: t(19) = 7.38, *p* < 0.001]. Within the BD group, NTS score was significantly correlated with RSQ scores in the anger domain (rho = –0.65, *p* = 0.012, q = 0.025, FDR corrected; Figure 1) and a near significant correlation was found between NTS and RSQ scores in the anxiety domain (rho = –0.55, *p* = 0.041, q = 0.05, FDR corrected). None of the correlations within the HC group were significant.

**TABLE 2:** Symptom severity and behavioral ratings for each group.

	Bipolar group Mean (*SE*)	Healthy group Mean (*SE*)	t (*df*)	Group comparison *p*-value
Depression (CDRS-R)	40.84 (2.93)	18.93 (0.47)	7.38 (18.94)	0.001*
Mania (YMRS)	7.42 (1.66)	2.14 (0.51)	3.04 (21.32)	0.006*
Subjective Distress (NTS)	2.87 (0.17)	3.16 (0.24)	1.01 (23.45)	0.325
Anger domain of the Rejection Sensitivity scale (RSQ)	9.64 (1.39)	5.40 (0.68)	2.73 (24.21)	0.012*
Anxiety domain of the Rejection Sensitivity scale (RSQ)	12.26 (1.49)	8.44 (0.97)	2.15 (27.92)	0.041*

SE, standard error; df, degrees of freedom; CDRS-R, Children’s Depression Rating Scale-Revised; YMRS, Young Mania Rating Scale; NTS, Need Threat Scale; RSQ, children’s Rejection Sensitivity Questionnaire. *Significant differences.

### fMRI Results

#### Group Differences in Activation to Exclusion vs Inclusion

**FIGURE 2:**
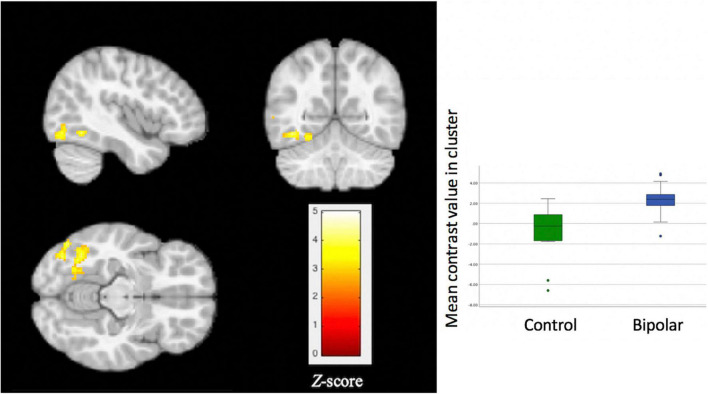
Significant group differences in activation of the left fusiform gyrus during a social exclusion task. Compared to healthy controls, youth with bipolar disorder showed significantly greater activation (*p* = 0.037) for the contrast of exclusion > inclusion. Thresholds for inference were set at *p* < 0.05, FWE corrected at the cluster level.

For the whole brain voxel-wise analysis, youth with BD showed significantly greater activation than HC in the left fusiform gyrus [FFG, Brodmann’s Area (BA) 37, peak X = –42, Y = –56, Z = –12, z = 3.71, cluster size = 270, *p* = 0.037, Figure 2]. For the ROI analysis, no significant differences were found between BD and HC for the ventral striatum ROI, ventral PFC ROI, and anterior insula ROI.

#### Functional Connectivity

**FIGURE 3:**
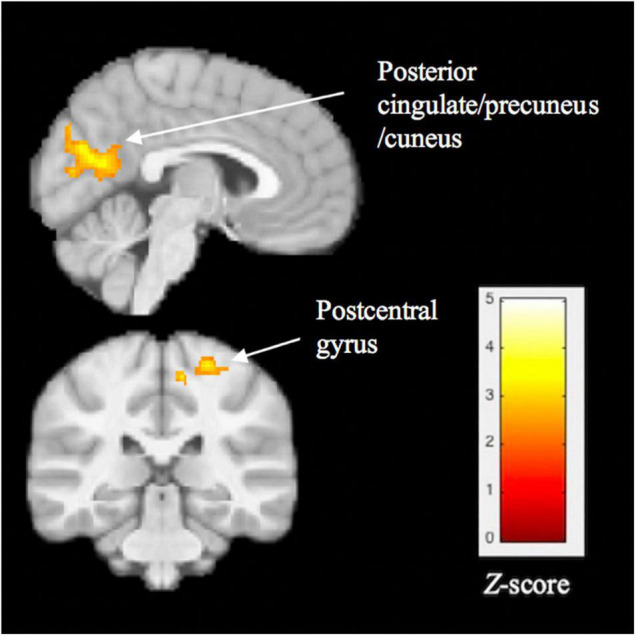
Significant group differences in task-related functional connectivity of the left fusiform gyrus, assessed using psychophysiological interaction analysis. Compared with healthy controls, youth with BD showed lower connectivity between the fusiform cluster and 3 regions: posterior cingulate, precuneus/cuneus, and postcentral gyrus. Thresholds were set at *p* < 0.05, FWE corrected at the cluster level.

The BD group, compared with the HC group, showed significantly lower functional connectivity between the left FFG cluster and two clusters: (1) posterior cingulate (PCC), precuneus, and cuneus (BA 23, 30, 31, 17 and 18; peak X = 6, Y = –66, Z = 18, z = 3.92, cluster size = 1617, *p* < 0.001) and (2) the postcentral gyrus (BA 3, 4; peak X = 24, Y = –32, Z = 64, z = 3.60, cluster size = 411, *p* = 0.006). These results are shown in Figure 3.

#### Associations Between Activation and Distress During Exclusion

**FIGURE 4:**
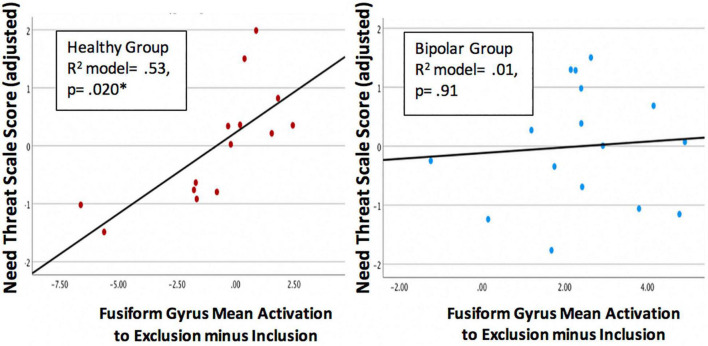
Scatterplots showing the association between activation of the left fusiform gyrus (FFG) and subjective distress during exclusion (Need Threat Scale total score adjusted for age) within the each group. Regression models were significant for the healthy control group (*p* = 0.020, **left**) but not the bipolar group (*p* = 0.91, **right**).

Within the HC group, fusiform gyrus activation was significantly associated with subjective distress during exclusion (total NTS score), after adjusting for age (model R square = 0.53, *p* = 0.020), such that lower distress during exclusion was associated with higher levels of FFG activation. Within the BD group, the association between subjective distress during exclusion and FFG activation was not significant (R square = 0.006, *p* = 0.961). A scatterplot of these associations is shown in Figure 4, for each group separately. No significant correlations were found between FFG activation and CDRS-R or YMRS scores within either group.

#### Whole Brain Associations Between Functional Connectivity and Distress During Exclusion

**FIGURE 5:**
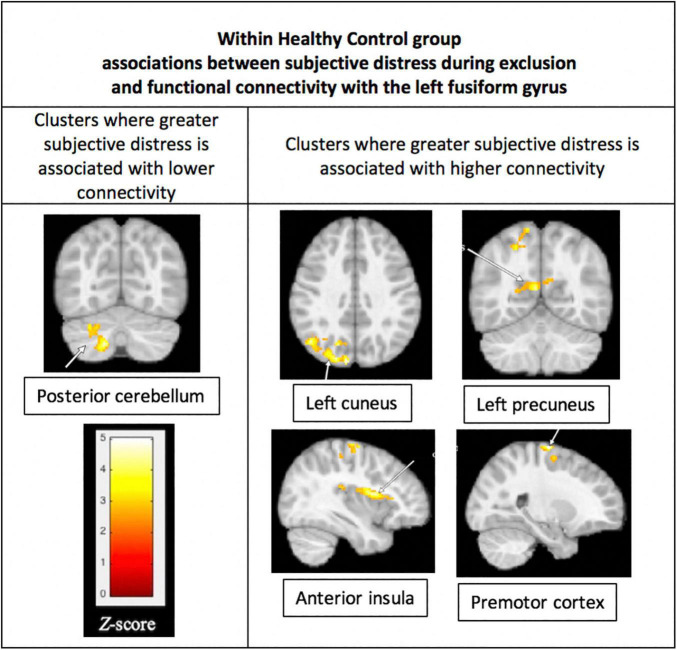
Associations between subjective distress and functional connectivity with the left fusiform gyrus (FFG). Within the HC group, greater distress during social exclusion was associated with lower connectivity between the fusiform gyrus and the left posterior cerebellum (*p* = 0.004), shown in the **(left)** column of the figure. Also for the HC group, greater distress was associated with higher connectivity of the cuneus, precuneus, insula, and premotor cortex, as shown in the **(right)** column. Functional connectivity was performed using the left FFG cluster as the seed in a generalized PPI analysis, with a threshold of *p* < 0.05, FWE corrected at the cluster level. Results were not significant within the Bipolar Disorder group.

Within the BD group, subjective distress during exclusion was not significantly associated with connectivity of the FFG. Within the HC group, greater distress during exclusion was significantly associated with lower connectivity between the left FFG and left posterior cerebellum (*p* = 0.004) and greater connectivity between the left FFG and four regions: (1) left cuneus (*p* < 0.001), (2) left precuneus (*p* = 0.008), (3) right anterior insula (*p* = 0.001), and (4) right premotor cortex (*p* = 0.001). These results are shown in Figure 5.

## Discussion

During a social exclusion task, youth with BD showed greater activation in the left FFG compared with HC. The HC group had greater functional connectivity over BD between the left FFG and the PCC compared to the BD group. Interestingly, there was no significant difference between the BD and HC groups in severity of distress during the social exclusion task. We found that distress significantly predicted FFG activation in the HC group but not in the BD group. In functional connectivity analysis for the HC group using the left FFG as the seed, greater connectivity with the right anterior insula, right premotor cortex, and left middle occipital cortex was also significantly correlated with greater feelings of distress. Taken together, these results suggest that youth with BD process social exclusion differently than healthy youth and that distress from social exclusion may correlate with alternate pathways not typically seen in healthy youth. These results are considered preliminary due to small sample size.

To our knowledge, this is the first fMRI study examining social exclusion in youth with BD. While we hypothesized we would see greater activation in the BD group, when compared with HC, in areas previously shown to hyperactivate in adolescent samples experiencing Cyberball, our study did not produce these results using ROI analysis. Regions in the HC group that correlated with greater distress in social exclusion, however, were the same that were hyperactivated in previous studies of Cyberball in healthy developmental samples (19). A recent meta-analysis of 53 cyberball neuroimaging studies including both adult and child samples, reported consistent recruitment of ventral anterior cingulate, posterior cingulate, inferior and superior frontal, insula and occipital cortex (34). These findings overlap with the 2017 meta-analysis (19) that also found consistent recruitment of the posterior cingulate and ventrolateral frontal corticies. While these meta-analyses do not include comparisons between clinical and healthy groups, they are relevant to the present findings of lower connectivity between posterior cingulate and fusiform gyrus in BD versus HC groups, suggesting that the fusiform is relevant to social exclusion through its connectivity to the posterior cingulate. We also note that the current findings of a correlation between subjective distress and functional connectivity of insula with fusiform gyrus within the healthy control group further suggest that the fusiform gyrus is clinically relevant because of its connectivity with regions that are consistently reported across previous studies of the cyberball paradigm.

To explore whether the BD group’s distress from social rejection was correlated with other brain regions, we conducted an exploratory whole brain voxel-wise correlation with NTS scores, but results were not significant. This could suggest the processing of distress after social rejection is not localized to a particular region or regions in the brain, or that our sample size was too small to detect an effect. However, the effect for the HC group was found with a smaller sample than the BD group.

The BD group did report some subjective distress but it was not significantly different than the HC group. However, the BD group had significantly higher ratings of anger and depression than the HC group, which were negatively correlated with subjective distress. This suggests that symptoms of anger and depression experienced by the BD group may have diminished or interfered with reporting subjective distress related to social exclusion in the BD group. If so, higher fusiform activation in BD may reflect an altered way of processing social exclusion that is not on a continuum with the HC group, e.g., not simply a more extreme level of subjective distress from social exclusion, but a different strategy, perhaps involving the visual system.

We hypothesized that the BD group would show greater activation in regions previously implicated in social exclusion in healthy controls (e.g., anterior cingulate cortex, VLPFC, and ventral striatum) but our results showed group differences in the fusiform gyrus. The fusiform gyrus is salient to higher level visual processing and implicated in facial perception, which are important components in the social cognitive circuit (35, 36). While some debate surrounds the function of the FFG, studies agree the area is recruited in the processing of faces (37). The role of facial processing and perception is important to understand the intention and emotions of others and therefore, to social interaction (37). In fact, a recent meta-analysis of the neural network of face processing in healthy adults showed the left posterior FFG was specifically involved in face processing tasks that required emotion evaluation (38). The posterior FFG, where our findings are located, encompasses the fusiform face area. Studies have shown this area to have higher activation when healthy adolescents are viewing fearful compared to neutral faces (39). Perhaps the BD group finds the depiction of cartoon faces in Cyberball emotionally evocative. This finding is consistent with previous literature suggesting adolescents with BD misinterpret neutral faces as fearful with greater hostility (40). Aberrant face emotion processing is well established in bipolar disorder, and so it is perhaps not surprising this marker of illness may be involved in any sort of social evaluative process (41).

Our connectivity analysis using the FFG as the seed showed the HC group had greater functional connectivity between the left FFG and the left PCC, specifically the caudal left PCC, which is an area associated with autobiographical memory. The left PCC is activated during successful autobiographical memory recollection in healthy adults (42). This may suggest that the HC group recalls social experiences more than the BD group in the context of social exclusion. The PCC is also implicated in tasks of emotional salience. Studies have shown hyperactivation of the PCC in tasks of both positive and negative emotional stimuli (43). These studies have postulated the strength of successful recall of autobiographical memories to be dependent on their emotional importance, and the PCC consistently hyperactivates on successful recall of such memories. This suggests the PCC moderates the interaction between memory and emotion (42). Healthy youth may be able to interpret social exclusion in the larger context of positive autobiographical memories of social experiences. Youth with BD, however, are known to have structural and functional abnormalities in the PCC, which may suggest they do not have the same ability to recall autobiographical experiences in the same way as healthy youth (44, 45).

Lastly, for the HC group only, lower distress during Cyberball was correlated with greater functional connectivity between the posterior cerebellum and the FFG. Studies suggest the posterior cerebellum connects with the limbic system and participates in the limbic related functions of emotion (46). The posterior cerebellum has been shown to have abnormal function and structure in youth with bipolar disorder (47). Lesions in the cerebellum have been implicated in causing manic states (48) and with problems with social interaction (49). It may be, then, that the HC group has a more intact emotional circuit during the social exclusion experience, unlike the BD group. Areas known to have structural and functional abnormalities in BD that overlap with our findings, specifically the PCC and the posterior cerebellum, are therefore associated with aberrant processing of social exclusion when compared with healthy youth.

Limitations of this study include a small sample size. However, this is the only published study to date to examine the functional neuroanatomy of youth with bipolar disorder using a social cognitive paradigm. The fMRI block design which provided only four minutes of game time data also is a limitation of this study. Current Cyberball fMRI studies have extended this model to provide more data points by using an alternating block design and multiple games in one scan (19). We did use FWE for fMRI analysis, which is a stringent thresholding method, but may have missed some relevant between group differences as a result. Future studies should examine whether domains of anxiety, affective lability, and coping skills moderate responses to social exclusion. We did find greater scores in the anger domain for the RSQ to significantly correlate with greater distress in the BD but not the HC group. A similar finding was discovered for the anxiety domain of the RSQ, though this finding did not reach significance. This suggests an emotional and anxious component in youth with BD that may predict the reaction to social exclusion that should be further explored.

In summary, despite aberrant neural processing, the BD group did not show significant differences in distress during social exclusion when compared with the HC group. Youth with BD may therefore process social exclusion in a manner different from the HC group that focuses on visual processes early in the social cognitive circuit while HC uses past social experiences to inform current social encounters. This difference in processing may pose clinical implications for improving social cognition in youth with BD and preventing mood symptoms.

## Data Availability Statement

Due to difficulties in de-identifying MRI images of this vulnerable child population, raw data are not available. Processed data are available upon request to the corresponding author, with a data sharing agreement.

## Ethics Statement

This study was reviewed and approved by the Institutional Review Board of Stanford University School of Medicine. Written informed assent and consent were provided by the youth and parent/guardian, respectively.

## Author Contributions

DR, KC, and AG designed the study. DR wrote the protocol and performed literature searches. DR, RML, and BN conducted the statistical analyses. DR, AG, RS, and RK conducted scans, pre-processed neuroimaging data, and analyzed neuroimaging data. All authors contributed to and approved the final manuscript.

## Conflict of Interest

The authors declare that the research was conducted in the absence of any commercial or financial relationships that could be construed as a potential conflict of interest.

## Publisher’s Note

All claims expressed in this article are solely those of the authors and do not necessarily represent those of their affiliated organizations, or those of the publisher, the editors and the reviewers. Any product that may be evaluated in this article, or claim that may be made by its manufacturer, is not guaranteed or endorsed by the publisher.

## Footnotes

1
http://www.fil.ion.ucl.ac.uk/spm

2
https://www.ibm.com/products/spss-statistics

3
http://marsbar.sourceforge.net/
